# Editorial: Advances in the cross-cultural assessment and diagnosis of developmental conditions

**DOI:** 10.3389/fpsyg.2023.1182767

**Published:** 2023-05-09

**Authors:** Themis Karaminis, Francesca Volpato, Stavroula Stavrakaki

**Affiliations:** ^1^Department of Psychology, Edge Hill University, Ormskirk, United Kingdom; ^2^Department of Linguistics and Comparative Cultural Studies, Ca' Foscari University of Venice, Venice, Italy; ^3^Department of Italian Language and Literature, Aristotle University of Thessaloniki, Thessaloniki, Greece

**Keywords:** neurodevelopmental disorders, cross-cultural/intercultural factors, cross-linguistic, diagnosis, assessment

Neurodevelopmental conditions, such as developmental language disorder (DLD), dyslexia, autism, and learning disabilities, affect how people perceive the world, think and learn, process information, and interact with each other throughout their lives. These conditions can have debilitating consequences in the everyday lives of the individuals affected by them, as well as their families. Early identification and diagnosis of neurodevelopmental conditions are essential for ensuring timely access to support and interventions and achieving better educational, health, and mental health outcomes (Elder et al., 2017; Lovett et al., 2017; Sansavini et al., 2021).

Over the past few decades, awareness of and research on neurodevelopmental disabilities have increased considerably. However, there are growing concerns that developmental disabilities research has been limited to a small number of cultural settings and languages, and, in particular, English-speaking environments (Bishop, 2010; Leonard, 1998/2014). This bias, which is similar to a more general bias of research toward WEIRD populations (Western, Educated, Industrialized, Rich, and Democratic; Henrich et al., 2010), challenges the identification and diagnosis of developmental conditions in some other languages and cultures, which are underrepresented in research.

One implication of this bias is that there are important asymmetries in the range and quality of care, support, and evidence-based interventions available to individuals with neurodevelopmental conditions across different countries or settings (e.g., Rinaldi et al., 2021), and, consequently, the ability of educators, clinicians, and researchers to work with these populations in different sociocultural settings.

Another implication is that our understanding of neurodevelopmental conditions is heavily skewed toward specific countries or cultures. Although it is recognized that language and culture affect the identification and diagnosis of neurodevelopmental conditions, we know little about these effects, which emerge from complex nature-nurture interactions (Mareschal et al., 2007; de Leeuw et al., 2020).

This Research Topic aims to address the following objectives. Firstly, we aim to present recent advances in diagnosing and identifying neurodevelopmental disorders in languages which are not well-represented in developmental research. Secondly, we aim to draw attention to the challenges and difficulties related to studying developmental conditions in these cultures. Finally, we aim to investigate implications from studies on languages belonging to different families, and thus showing different surface properties, for the diagnosis of individuals with neurodevelopmental conditions. The scope of the topic also includes studies related to the cultural adaptation and validation of assessment tools, and the development of new assessment tools.

We accepted five submissions for publication, four focusing on Greek and one on Mandarin. Greek is spoken by around 13.5 million people (Ethnologue; Eberhard et al., 2021) in Greece, Cyprus, and the diaspora. It is a language with an especially rich morphology, which allows for flexible word order, and a relatively transparent orthographic system. Mandarin is spoken by around 1.1 billion people in China as well as in Taiwan, Malaysia, Indonesia, and Singapore (Ethnologue; Eberhard et al., 2021). It falls in the category of analytic languages, which convey relationships between words in sentences primarily using helper words (particles, prepositions, etc.) and word order. Mandarin has a monosyllabic writing system as a basic orthographic unit (i.e., character) that typically corresponds to one syllable and one morpheme (Hoosain, 1992).

Antoniou et al. present important findings from a large-scale study examining the administration of Logometro^®^to 926 children between the ages of 4 and 7. Of the participants, 800 were typically developing, and 126 had a diagnosis of DLD. This is a significant sample size in the context of current research in Greek. Logometro is a comprehensive tool that evaluates a range of oral language skills, including phonological awareness, listening comprehension, vocabulary knowledge (receptive and expressive), narrative speech, morphological awareness, pragmatics, as well as emergent literacy skills such as letter sound knowledge and invented writing. The results from Antoniou et al.'s study are valuable as they map developmental trajectories and provide comprehensive developmental language profiles. Additionally, they demonstrate the usefulness of Logometro in diagnosing DLD and describe the characteristics of DLD in Greek.

Helidoni et al. focus on early lexical development, which they assessed using the adapted Cyprus Greek Lexical List (a-CYLEX GR). The a-CYLEX GR is a checklist based on parental reports and provides an assessment of early receptive and expressive vocabulary skills in children between the ages of 12 months and 3 years and 6 months. Helidoni et al. report findings from a sample of 194 Greek toddlers and demonstrate that this checklist fulfills psychometric criteria for validity and reliability. The authors also investigate the impact of demographic factors on vocabulary development and discuss them in light of previous findings. Employing the a-CYLEX GR will significantly contribute to everyday clinical practice and research projects, particularly since this checklist provides an assessment of the very early vocabulary abilities of Greek-speaking children.

Talli et al. provide a task for assessing phonological short-term memory in Greek-speaking individuals, which is an essential component of language and is implicated in difficulties associated with language disorders (Gathercole, 2006; Gathercole and Alloway, 2006). Notably, deficits in non-word repetition (NWR) have been considered, cross-linguistically, as a clinical marker for DLD (see, among others, Bishop et al., 1996; Thordardottir and Reid, 2022). To contribute to accurate assessment and clinical diagnosis, Talli et al. investigated the reliability and validity of an NWR task in a large cohort of 387 TD Greek-speaking children between the ages of 7 and 13. The results of this study suggest that this NWT could function as a reliable and valid measure of phonological short-term memory (STM). Additionally, they show that while phonological STM increases in capacity with age, it does not increase after the age of 10. Overall, the study by Talli et al. indicates that this task could be employed in both clinical and research contexts as an accurate measure of phonological STM.

Next, we turn to written language. Andreou and Aslanoglou offer cross-linguistic insights into the written language difficulties of a group of 31 children with DLD compared to a group of 31 typically developing (TD) children. Unlike English, in which orthography is opaque for both reading and spelling, Greek is a language in which orthography is transparent for reading but opaque for spelling. The results of Andreou and Aslanoglou's study for Greek suggest that it is necessary to assess written language production in addition to oral language in Greek-speaking children with DLD. This finding is significant for the cross-linguistic assessment of written language difficulties and emphasizes the importance of assessing written language in addition to oral language.

Xie et al. have made an additional contribution to the assessment of written language by focusing on Mandarin. Orthographic knowledge awareness is crucial for the development of reading and spelling skills, while children lacking this ability may show learning difficulties. Xie et al. addressed, exactly, the lack of comprehensive tools for assessing orthographic knowledge in Mandarin. They created an evaluation scale based on a literature review and qualitative interviews and consultations with experts, and validated it using data collected from 1,354 questionnaires completed by children attending primary schools in different areas of Jiangsu Province. This tool can aid in the early detection of reading and writing difficulties in 6- to 12-year-old children, and can facilitate the development of intervention approaches to improve impaired skills.

This Research Topic contributes to the field of cross-linguistic assessment of neurodevelopmental conditions in three significant ways. Firstly, it presents new tools for assessing oral and written language development in two languages that have been underrepresented in research on neurodevelopmental disorders and atypical language development. The assessment materials are validated and proven reliable, thus constituting valuable resources for identifying and diagnosing developmental conditions in these two languages. Furthermore, these tools could be employed in future research to determine the prevalence of developmental conditions and characterize the manifestation of developmental and language-related conditions in these languages.

Secondly, the Research Topic sheds light on several challenges encountered when studying developmental conditions in Greek and Mandarin, which are relevant to research in other underrepresented languages. All the studies emphasized the scarcity of relevant diagnostic tools and resources. In addition, the study on orthographic awareness in Mandarin revealed an additional challenge due to the typologically rare orthography of the language. Consequently, the authors had to develop a new tool, the orthographic knowledge awareness scale, rather than simply adapting an existing one. Another challenge encountered was the smaller sample size in the studies involving Greek children, possibly reflecting the limited size of the population and potential limitations in infrastructure and awareness.

Finally, the Research Topic underscores implications of studying developmental conditions in languages with different surface properties than those commonly examined. Findings have broad-ranging implications for the diagnosis and the understanding of developmental conditions beyond the two languages studied in the contributions of this collection. For example, Andreou and Aslanoglou emphasize the importance of assessing both oral and written language skills in children with DLD, especially in languages featuring opaque writing systems. Similarly, Xie et al. identify processes that are critical for the acquisition of orthographic knowledge in languages without alphabetic scripts. Further research is warranted to determine the extent to which these findings generalize to languages with similar typological characteristics and to account for developmental patterns cross-linguistically. This would be a significant step toward addressing the challenge delineating complex nature-nurture interactions underlying individual developmental differences (Mareschal et al., 2007).

## Author contributions

All authors contributed to editing the Research Topic and authoring the editorial.
